# Supplementary material for: Modeling glioblastoma heterogeneity as a dynamic network of cell states
Source: Mol Syst Biol. 2021 Sep 16;17(9):e10105. doi: 10.15252/msb.202010105 (PMC8444284; doi:10.15252/msb.202010105)
Supplement: Supplementary file 6 — Source Data for Figure 5 [file MSB-17-e10105-s004.zip › Figure5A_sourcedata/GSEA_3071/hallmarks_stateI.GseaPreranked.1623742531165/HALLMARK_COMPLEMENT.html]

Details for gene set HALLMARK\_COMPLEMENT[GSEA]

|  || Dataset | state13071 |
| Phenotype | NoPhenotypeAvailable |
| Upregulated in class | na\_pos |
| GeneSet | HALLMARK\_COMPLEMENT |
| Enrichment Score (ES) | 0.36870927 |
| Normalized Enrichment Score (NES) | 1.9441688 |
| Nominal p-value | 0.0 |
| FDR q-value | 0.005339656 |
| FWER p-Value | 0.032 |
Table: GSEA Results Summary

  

Fig 1: Enrichment plot: HALLMARK\_COMPLEMENT      
 Profile of the Running ES Score & Positions of GeneSet Members on the Rank Ordered List

  

| PROBE | GENE SYMBOL | GENE\_TITLE | RANK IN GENE LIST | RANK METRIC SCORE | RUNNING ES | CORE ENRICHMENT || 1 | FN1 |  |  | 11 | 1.769 | 0.0453 | Yes |
| 2 | MMP14 |  |  | 27 | 1.526 | 0.0828 | Yes |
| 3 | CFH |  |  | 29 | 1.523 | 0.1242 | Yes |
| 4 | TIMP1 |  |  | 47 | 1.381 | 0.1572 | Yes |
| 5 | COL4A2 |  |  | 73 | 1.203 | 0.1829 | Yes |
| 6 | LRP1 |  |  | 84 | 1.160 | 0.2118 | Yes |
| 7 | CTSB |  |  | 129 | 0.964 | 0.2255 | Yes |
| 8 | CEBPB |  |  | 222 | 0.782 | 0.2203 | Yes |
| 9 | IRF1 |  |  | 276 | 0.705 | 0.2242 | Yes |
| 10 | S100A13 |  |  | 306 | 0.665 | 0.2341 | Yes |
| 11 | HSPA5 |  |  | 337 | 0.638 | 0.2429 | Yes |
| 12 | LAMP2 |  |  | 352 | 0.629 | 0.2560 | Yes |
| 13 | DYRK2 |  |  | 353 | 0.628 | 0.2732 | Yes |
| 14 | LGALS3 |  |  | 390 | 0.600 | 0.2793 | Yes |
| 15 | ANXA5 |  |  | 392 | 0.597 | 0.2953 | Yes |
| 16 | PSMB9 |  |  | 437 | 0.560 | 0.2979 | Yes |
| 17 | CTSL |  |  | 440 | 0.558 | 0.3126 | Yes |
| 18 | CTSO |  |  | 472 | 0.532 | 0.3183 | Yes |
| 19 | CTSD |  |  | 474 | 0.531 | 0.3325 | Yes |
| 20 | CPQ |  |  | 488 | 0.523 | 0.3431 | Yes |
| 21 | CTSS |  |  | 509 | 0.502 | 0.3510 | Yes |
| 22 | ME1 |  |  | 543 | 0.484 | 0.3547 | Yes |
| 23 | HSPA1A |  |  | 566 | 0.473 | 0.3613 | Yes |
| 24 | CASP4 |  |  | 585 | 0.460 | 0.3687 | Yes |
| 25 | LIPA |  |  | 700 | 0.412 | 0.3470 | No |
| 26 | CD55 |  |  | 726 | 0.403 | 0.3508 | No |
| 27 | PCLO |  |  | 860 | 0.362 | 0.3222 | No |
| 28 | JAK2 |  |  | 886 | 0.353 | 0.3246 | No |
| 29 | CSRP1 |  |  | 921 | 0.345 | 0.3242 | No |
| 30 | DOCK10 |  |  | 957 | 0.338 | 0.3234 | No |
| 31 | CASP1 |  |  | 981 | 0.331 | 0.3258 | No |
| 32 | LGMN |  |  | 1031 | 0.321 | 0.3204 | No |
| 33 | ERAP2 |  |  | 1240 | 0.280 | 0.2678 | No |
| 34 | GZMB |  |  | 1273 | 0.274 | 0.2660 | No |
| 35 | CD46 |  |  | 1355 | 0.259 | 0.2497 | No |
| 36 | CBLB |  |  | 1398 | 0.252 | 0.2444 | No |
| 37 | GATA3 |  |  | 1417 | -0.250 | 0.2461 | No |
| 38 | GMFB |  |  | 1463 | -0.254 | 0.2400 | No |
| 39 | CASP3 |  |  | 1639 | -0.273 | 0.1968 | No |
| 40 | RABIF |  |  | 1772 | -0.290 | 0.1665 | No |
| 41 | CALM1 |  |  | 1783 | -0.291 | 0.1716 | No |
| 42 | CDK5R1 |  |  | 1879 | -0.302 | 0.1523 | No |
| 43 | CTSC |  |  | 1975 | -0.315 | 0.1334 | No |
| 44 | SCG3 |  |  | 2025 | -0.322 | 0.1280 | No |
| 45 | ADAM9 |  |  | 2063 | -0.328 | 0.1263 | No |
| 46 | MT3 |  |  | 2179 | -0.348 | 0.1025 | No |
| 47 | RAF1 |  |  | 2213 | -0.356 | 0.1027 | No |
| 48 | PFN1 |  |  | 2305 | -0.373 | 0.0866 | No |
| 49 | PPP2CB |  |  | 2384 | -0.390 | 0.0747 | No |
| 50 | PLAT |  |  | 2501 | -0.415 | 0.0525 | No |
| 51 | CALM3 |  |  | 2744 | -0.484 | -0.0044 | No |
| 52 | GNAI2 |  |  | 2889 | -0.535 | -0.0315 | No |
| 53 | PRCP |  |  | 2919 | -0.553 | -0.0247 | No |
| 54 | GNB4 |  |  | 2943 | -0.568 | -0.0158 | No |
| 55 | SERPINE1 |  |  | 3050 | -0.634 | -0.0291 | No |
| 56 | DUSP6 |  |  | 3227 | -0.816 | -0.0577 | No |
| 57 | PDP1 |  |  | 3376 | -1.117 | -0.0700 | No |
| 58 | CDH13 |  |  | 3414 | -1.277 | -0.0457 | No |
| 59 | CLU |  |  | 3424 | -1.329 | -0.0119 | No |
| 60 | PDGFB |  |  | 3428 | -1.343 | 0.0240 | No |
Table: GSEA details [plain text format]

  

Fig 2: HALLMARK\_COMPLEMENT: Random ES distribution      
 Gene set null distribution of ES for **HALLMARK\_COMPLEMENT**
